# Measuring adherence to antiretroviral therapy in children and adolescents in western Kenya

**DOI:** 10.7448/IAS.17.1.19227

**Published:** 2014-11-25

**Authors:** Rachel C Vreeman, Winstone M Nyandiko, Hai Liu, Wanzhu Tu, Michael L Scanlon, James E Slaven, Samuel O Ayaya, Thomas S Inui

**Affiliations:** 1 Children's Health Services Research, Department of Pediatrics, Indiana University School of Medicine Indianapolis, IN, USA; 2 Academic Model Providing Access to Healthcare (AMPATH), Eldoret, Kenya; 3 Regenstrief Institute, Inc., Indianapolis, IN, USA; 4 Department of Child Health and Paediatrics, Moi University School of Medicine, Eldoret, Kenya; 5 Department of Biostatistics, Indiana University School of Medicine Indianapolis, IN, USA; 6 Department of Medicine, Indiana University School of Medicine Indianapolis, IN, USA

**Keywords:** adherence, paediatric HIV, best practice, resource-limited setting

## Abstract

**Introduction:**

High levels of adherence to antiretroviral therapy (ART) are central to HIV management. The objective of this study was to compare multiple measures of adherence and investigate factors associated with adherence among HIV-infected children in western Kenya.

**Methods:**

We evaluated ART adherence prospectively for six months among HIV-infected children aged ≤14 years attending a large outpatient HIV clinic in Kenya. Adherence was reported using caregiver report, plasma drug concentrations and Medication Event Monitoring Systems (MEMS^®^). Kappa statistics were used to compare adherence estimates with MEMS^®^. Logistic regression analyses were performed to assess the association between child, caregiver and household characteristics with dichotomized adherence (MEMS^®^ adherence ≥90% vs. <90%) and MEMS^®^ treatment interruptions of ≥48 hours. Odds ratios (ORs) and 95% confidence intervals (95% CIs) were calculated.

**Results:**

Among 191 children, mean age at baseline was 8.2 years and 55% were female. Median adherence by MEMS^®^ was 96.3% and improved over the course of follow-up (*p*<0.01), although 49.5% of children had at least one MEMS^®^ treatment interruption of ≥48 hours. Adherence estimates were highest by caregiver report, and there was poor agreement between MEMS^®^ and other adherence measures (Kappa statistics 0.04–0.37). In multivariable logistic regression, only caregiver-reported missed doses in the past 30 days (OR 1.25, 95% CI 1.14–1.39), late doses in the past seven days (OR 1.14, 95% CI 1.05–1.22) and caregiver-reported problems with getting the child to take ART (OR 1.10, 95% CI 1.01–1.20) were significantly associated with dichotomized MEMS^®^ adherence. The caregivers reporting that ART made the child sick (OR 1.12, 95% CI 1.01–1.25) and reporting difficulties in the community that made giving ART more difficult (e.g. stigma) (OR 1.14, 95% CI 1.02–1.27) were significantly associated with MEMS^®^ treatment interruptions in multivariable logistic regression.

**Conclusions:**

Non-adherence in the form of missed and late doses, treatment interruptions of more than 48 hours and sub-therapeutic drug levels were common in this cohort. Adherence varied significantly by adherence measure, suggesting that additional validation of adherence measures is needed. Few factors were consistently associated with non-adherence or treatment interruptions.

## Introduction

The advent of antiretroviral therapy (ART) has significantly improved the long-term survival of HIV-infected children [1–5], and good outcomes rely on high rates of adherence to therapy [6–9]. The International Association of Physicians in AIDS Care recommends routine monitoring of adherence to ART for all patients in clinical settings to guide clinical decision making, prevent drug resistance, and evaluate adherence interventions, but there are no specific recommendations for monitoring adherence in paediatric HIV [10].

Recent studies suggest that children in low- and middle-income countries (LMICs) have similar or better rates of ART adherence compared to children from high-income countries; however, most estimates of paediatric adherence in LMICs come from heterogeneous and unvalidated measures [11–13]. Adherence assessment by caregiver reports is commonly used in studies reporting ART among children and is consistently higher than adherence by other measures like pill count or pharmacy refill, suggesting that caregivers likely overestimate their child's adherence [12,13]. Electronic dose monitoring with technologies like Medication Event Monitoring Systems (MEMS^®^) – bottle caps fitted with a microchip that records the time and date of each bottle opening – is most consistently associated with virological outcomes [14,15] but is often too expensive for routine use in LMICs outside of research settings [16].

For children in LMICs who have limited access to second- and third-line treatment options, the preservation of first-line regimens through high rates of adherence is particularly important for survival into adolescence and adulthood [17,18]. Despite the importance of adherence to ART, children in LMICs often face multiple and complex barriers to achieving optimal adherence, and there are few data to inform adherence interventions [19]. A better understanding of factors that are associated with adherence in HIV-infected children is critical to the design and implementation of effective interventions.

The Academic Model Providing Access to Healthcare (AMPATH) provides comprehensive HIV care for over 5000 HIV-infected children (<15 years of age) on ART in western Kenya. We conducted a cohort study to describe adherence to ART among children in this setting. Our objective was twofold: 1) to describe adherence to ART using multiple measures and compare routine measures (e.g. caregiver-reported adherence) to MEMS^®^, and 2) investigate factors associated with poor adherence. This study adds to the literature on best practices for measuring adherence to ART among HIV-infected children in LMICs, and it may be used to inform adherence interventions in this population.

## Methods

### Study design

We conducted a prospective cohort study with 200 HIV-infected children, ≤14 years of age and on ART and their caregivers at AMPATH's largest paediatric clinic, which is located at Moi Teaching and Referral Hospital (MTRH) in Eldoret – the fifth largest city in Kenya. Participants were followed for six months and participated in monthly study visits, which took place in a private room with study personnel. Study visits were scheduled on the same day as the child's routine clinical visit; after the child was seen by their regular care provider, the child and caregiver would come to the research office for their study visit. Demographic and clinical characteristics were extracted by chart review. The study was approved by the Institutional Review Board at Indiana University School of Medicine in Indianapolis, Indiana, USA and by the Institutional Research and Ethics Committee at Moi University School of Medicine in Eldoret, Kenya.

### Setting

AMPATH is a partnership between Moi University School of Medicine, MTRH and a consortium of North American academic medical centres led by Indiana University School of Medicine. AMPATH provides free ART (first- and second-line ART regimens only), primary care services and psychosocial and nutritional support for children and adults.

### Study participants

A convenience sample of 200 caregiver-child dyads were identified by clinic and study personnel and referred for study participation. The target sample size for this study was selected to enable confident testing of up to 40 adherence questionnaire items, which is reported elsewhere. For psychometric analyses, at least five subjects per one item (with no fewer than 100 participants) are considered necessary for factor analysis [20]. Additionally, this sample size gave us adequate power to detect a beta coefficient of 0.4 at a 0.05 significance level under the conservative assumption of an error variance of 4 and independent variable standard deviation of 1.

Eligible children were HIV-infected, ≤14 years old, on either a nevirapine (NVP)- or efavirenz (EFV)-based first-line ART regimen and enrolled in care at the AMPATH paediatric outpatient clinic at MTRH. Fourteen was the maximum age limit for enrolment because children older than this age are routinely treated in the adult clinic. Enrolment was limited to children on first-line ART containing NVP or EFV because plasma drug concentrations were only available for these drugs, and NVP or EFV is part of the standard first-line paediatric regimen at AMPATH. The child's current level of adherence was not considered for study referral or selection; however, it is possible that patients who had higher or lower levels of adherence would be more likely to enrol. “Caregiver” was defined as an individual who both accompanied the child to clinical and study visits and had knowledge of the child's medication taking. While we encouraged the same caregiver to come to all assessments, we did not exclude different caregivers (e.g. mother versus grandfather) from participating in the study assessments. Informed consent was obtained for all caregivers, and assent for any child 10 years and older, in line with standard AMPATH research protocols. A small incentive was provided for participation to help cover transportation costs and time.

### Adherence measures

Monthly adherence assessments included caregiver-reported adherence, drug concentrations and electronic medication monitoring using MEMS^®^ (AARDEX, Inc.). Caregiver-reported adherence was assessed through a 48-item questionnaire that included questions about missed or late doses, adherence barriers, household characteristics and a visual analogue scale (VAS) (adherence questionnaire provided under “Supplementary File”). VAS was used to assess the number of doses the child took over the last 30 days, with the caregivers indicating doses taken on a horizontal line; the rightmost side indicated that all doses were taken, and the leftmost side indicated no doses taken. A trained research assistant administered the questionnaire items verbally in Kiswahili or English (depending on the caregiver's language preference) and then recorded the caregivers’ verbal responses on a paper form.

The patients’ NVP or EFV was kept in a bottle with a MEMS^®^ cap for continuous electronic monitoring of medication dose timing throughout the study period. At enrolment, patients were informed of the purpose of the MEMS^®^ cap and instructed in care of the cap and bottle. At each visit, study staff downloaded data from the MEMS^®^ caps and shared these data with the caregivers and children by showing them the computer display with the record of dose timing using PowerView software (Version 3.5.2; AARDEX, Inc.). The downloading and sharing of dose timing from MEMS^®^ were conducted after the administration of the adherence questionnaires at every visit. Replacement MEMS^®^ were given to patients who reported damaged or lost MEMS^®^. MEMS^®^ events that occurred during the study assessments (e.g. opening MEMS^®^ to conduct pill counts) were censored for analyses.

NVP and EFV drug concentrations were taken at two time points (month 1 and month 4). Drug concentrations are not available routinely at AMPATH, but were run for study purposes in the AMPATH Reference Laboratory in Eldoret using a rapid, automated enzyme immunoassay developed by ARK Diagnostics (Sunnyvale, CA, USA). The ARK NVP-Test and ARK EFV-Test are based on competitive binding to antibody between the drug in the sample and a drug-labelled enzyme. Drug concentration was measured spectrophotometrically in terms of enzyme activity.

In the clinical care setting at AMPATH, the only routine adherence measure for children consists of clinicians asking either the caregiver or the child the following two questions: “During the last month, has the patient missed any medications?” and “During the last seven days, how many pills did the patient take?” These adherence data were extracted for study participants by chart review and compared to study-administered adherence measures.

### Data analysis

Adherence by caregiver-reported missed doses and VAS adherence were dichotomized into “adherent” (defined as no indication of missed doses in the recall period) versus “non-adherent” (defined as any indication of missed doses) at each visit. Any indication of non-adherence was categorized as “non-adherent” because reports of non-adherence were so scarce using these measures and caregiver reports generally overestimate actual adherence [21]. Plasma drug concentrations were categorized into therapeutic levels: sub-therapeutic (NVP <3.0 µg/mL or EFV <1.0 µg/mL), therapeutic (NVP 3.0–7.6 µg/mL or EFV 1.0–4.0 µg/mL) or supra-therapeutic (NVP >7.6 µg/mL or EFV >4.0 µg/mL) [22,23]. Adherence by plasma drug concentration was dichotomized as “adherent” (defined as therapeutic or supra-therapeutic) versus “non-adherent” (defined as a sub-therapeutic range). For MEMS^®^ adherence, mean and median adherence levels were calculated by visit and across visits to estimate the percentage of doses of NVP or EFV taken. MEMS^®^ adherence was also dichotomized as “adherent” (defined as ≥90% of doses taken by MEMS^®^) versus “non-adherent” (defined as <90% of doses taken). While we recognize that 90% adherence may not always be sufficient for viral suppression, MEMS^®^ was dichotomized at above or below 90% adherence for consistency with previous studies [24,25]. Furthermore, studies show that below this level of adherence, the risks for HIV virological rebound and drug resistance are increased, particularly for older non-nucleoside reverse transcriptase inhibitors like EFV and NVP [26–28].

Univariable analyses with Student's *t*-tests and Pearson's chi-square tests were used to explore relationships between dichotomized MEMS^®^ adherence and other adherence measures as well as demographic, clinical and psychosocial characteristics of the child and caregiver. Repeated-measures logistic regression analyses using odds ratios (ORs) and 95% confidence intervals (95% CIs) with and without adjusting for baseline characteristics (gender, age and duration on ART) were conducted using adherence data from each monthly visit. Due to the high correlation between caregiver-reported missed-doses variables using different recall periods (three-day, seven-day and 30-day), inclusion into the multivariable model was restricted to only one of these. The 30-day missed-dose variable was chosen as it had the smallest *p*-value in univariable models. We also used univariable and multivariable analyses (with and without adjusting for baseline characteristics, as above) to investigate factors associated with MEMS^®^ treatment interruptions, defined as a single period of 48 hours or greater with no recorded bottle opening. Kappa statistics were calculated to compare adherence estimates by the different methods of measurement to MEMS^®^. We used MEMS^®^ as the comparison adherence measure because it is commonly used as the reference standard in studies using multiple measures of adherence and best correlates with virologic outcomes [29–31]. All statistical analyses were performed using SAS Version 9.3 (SAS Institute, Cary, NC).

## Results

### Study participant characteristics

Among 191 caregiver-child dyads followed for six months, mean age of children at baseline was 8.2 years and 55% were female. Weight-for-age Z (WAZ) scores indicated mild malnutrition, with a mean WAZ score of −1.7. Mean duration on ART was 2.3 years, with most children on NVP-based regimens (77%). Children in this cohort had significant disease progression, with 54% at World Health Organization (WHO) Stage 3 at study start and a mean CD4 percentage of 26%. The most common type of caregiver participant was the biological mother of the child (63%), but there were also a number of father (11%) and grandparent (7%) participants. For the vast majority of children (90%), the same caregiver was present for all adherence assessments during the study period. Caregivers reported high levels of food insecurity (68%) and difficulties with transportation to clinic (84%). There were 17 participants (9%) who had to have their MEMS^®^ replaced during the study period due to damage or MEMS^®^ not functioning properly. In univariable analyses, there was no significant difference in baseline demographic and clinical factors between children who were always adherent during the study period (MEMS^®^ adherence at every month >90%) and children who were non-adherent at least once (MEMS^®^ adherence <90% for at least one month) (Table 1).

**Table 1:** Caregiver-child dyad characteristics by MEMS^®^ adherent group

	Overall	Adherent (MEMS^®^ >90% doses taken at every visit)	Ever non-adherent (MEMS^®^ <90% doses taken at any visit)	
		
Characteristic	*N*=191	*N*=56	*N*=134	*p*
Child characteristics				
Mean age (years)	8.2 (3.3)	8.3 (3.1)	8.1 (3.3)	0.70
Female	105 (55%)	29 (52%)	76 (57%)	0.53
Mean weight-for-age Z (WAZ) score	−1.7 (1.3)^a^	−1.7 (1.2)	−1.7 (1.3)	0.80
Mean ART duration (years)	2.3 (1.9)	2.6 (1.8)	2.2 (1.8)	0.18
ART regimen				
NVP	148 (77%)	47 (84%)	101 (74%)	0.26
EFV	43 (22%)	9 (16%)	34 (25%)	
NVP/EFV (both)	2 (1%)	0 (0%)	2 (1%)	
Mean CD4%	26% (11%)	30% (9%)	25% (11%)	0.06
WHO Stage				
1	34 (18%)	11 (20%)	23 (17%)	0.50
2	32 (17%)	11 (20%)	20 (15%)	
3	104 (54%)	31 (55%)	75 (55%)	
4	18 (9%)	3 (5%)	15 (11%)	
Not answered	3 (2%)	0 (0%)	3 (2%)	
Disclosure status				
Child disclosed	44 (23%)	10 (18%)	34 (25%)	0.26
Caregiver and/or family characteristics				
Caregiver relationship to child				
Mother	121 (63%)	32 (57%)	88 (66%)	0.14
Father	21 (11%)	7 (13%)	14 (10%)	
Sibling	3 (2%)	0 (0%)	3 (2%)	
Grandparent	13 (7%)	3 (5%)	10 (7%)	
Non-relative	7 (4%)	5 (9%)	2 (1%)	
Other	26 (14%)	9 (16%)	17 (13%)	
Individuals who give the child ART^b^				
Mother	160 (84%)	45 (80%)	114 (85%)	0.42
Father	68 (36%)	21 (38%)	46 (34%)	0.68
Sibling	80 (42%)	18 (32%)	62 (46%)	0.07
Other relative	68 (36%)	20 (36%)	48 (36%)	0.98
Child took own	60 (31%)	13 (23%)	47 (35%)	0.11
Caregiver employed outside the home	99 (52%)	27 (49%)	72 (54%)	0.56
Enrolled in AMPATH nutrition programme	33 (17%)	8 (14%)	24 (18%)	0.54
Food insecurity (reported “not enough food for family”)	135 (68%)	36 (64%)	98 (73%)	0.22
Reported difficulty with transportation to clinic	159 (84%)	44 (79%)	114 (86%)	0.23

a WHO classifies −2<WAZ Score<−1 as “mild malnourishment”

b rows do not sum to 100% because participants could report multiple persons who gave medicines.

### Adherence by multiple measures

**Figure 1:**
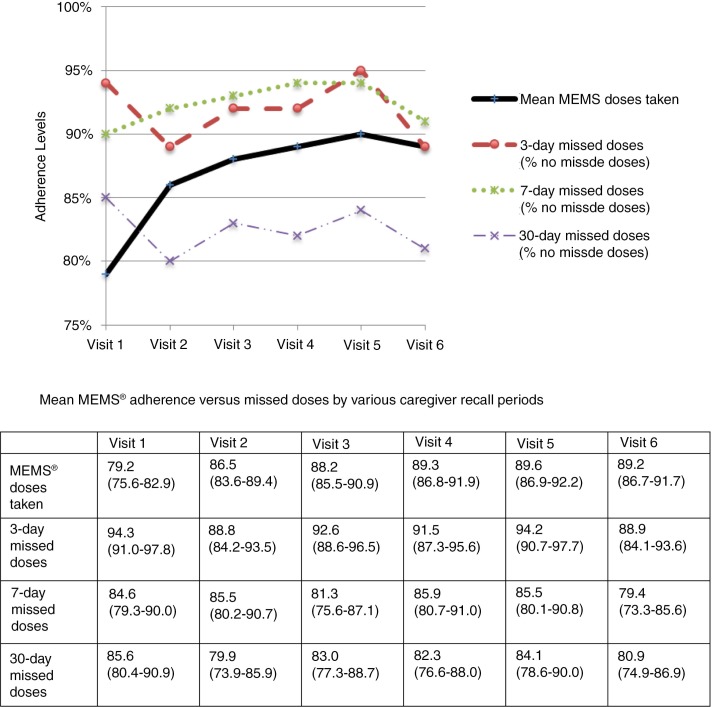
Values: Mean adherence by multiple measures with 95% confident intervals

Mean adherence by MEMS^®^ was 87% (median adherence by MEMS^®^ was 96%) and improved significantly over the course of the study; while only 51% achieved MEMS^®^ adherence ≥90% at month 1, 70% did so by month 6. Treatment interruptions were common; 49% of children had at least one MEMS^®^ treatment interruption of ≥48 hours (with a median of 3 MEMS^®^ treatment interruptions per child over the course of the study). Adherence by caregiver-reported missed doses was higher using the three-day recall item (92% reported no missed doses) and seven-day recall (92% reported no missed doses) compared to the 30-day recall item (83% reported no missed doses). Caregivers reported even higher adherence by VAS (94% reported no missed doses). In contrast to MEMS^®^, caregiver-reported missed doses generally showed consistent or decreasing adherence across the study period (Figure 1). Fourteen per cent of children on NVP and 27% on EFV had sub-therapeutic drug levels, whereas 59% of children on NVP and 23% on EFV had supra-therapeutic drug levels (Table 2). Caregiver-reported missed doses to clinicians at routine clinic visits suggested the highest rates of adherence (97% reported no missed doses).

**Table 2:** Adherence levels using multiple measures by visit

Adherence measure	Visit 1	Visit 2	Visit 3	Visit 4	Visit 5	Visit 6	Composite
MEMS^®^ measures							
Mean MEMS^®^ doses taken (SD)	79% (26%)	86% (21%)	88% (19%)	89% (18%)	90% (19%)	89% (18%)	87%
Median MEMS^®^ doses taken (IQR)	93% (73–96)	96% (82–100)	96% (89–100)	96% (88–100)	96% (87–100)	96% (85–100)	96%
Dichotomized MEMS^®^ (≥90% doses taken)	51%	64%	67%	67%	68%	68%	68%
Caregiver-reported measures							
3-day: missed doses (% no missed doses)	94%	89%	92%	92%	95%	89%	92%
7-day: missed at least one dose in a day (% no missed doses)	85%	85%	81%	86%	85%	79%	84%
7-day: missed all doses in a day (% no missed doses)	90%	92%	93%	94%	94%	91%	92%
7-day: late dose in a day (% no missed doses)	75%	74%	73%	72%	74%	73%	74%
30-day: missed doses (% no missed doses)	85%	80%	83%	82%	84%	81%	83%
Dichotomized VAS (% no missed morning doses)	96%	91%	95%	94%	93%	91%	93%
Dichotomized VAS (% no missed evening doses)	99%	99%	93%	96%	92%	91%	95%
Drug concentration measures							
NVP plasma							
Sub-therapeutic	15%			12%			n/a
Optimal	34%			22%			
Supra-therapeutic	51%			66%			
EFV plasma							
Sub-therapeutic	21%			32%			n/a
Optimal	55%			46%			
Supra-therapeutic	24%			22%			
Routine AMPATH adherence							
AMPATH Clinical Encounter Form (% no missed doses in past 30 days)	98%	96%	95%	99%	96%	96%	97%

### Agreement between adherence measures

There was poor agreement between dichotomized MEMS^®^ and other adherence measures, but most measures did show a statistically significant association with MEMS^®^ (Table 3). Among the study-administered measures, missed doses by seven-day recall (range of Kappa statistic: 0.11 to 0.34) and 30-day recall (range: 0.10 to 0.37) had highest agreement with the MEMS^®^. Missed doses by three-day recall (range: 0.05 to 0.25) had lower agreement with dichotomized MEMS^®^, and VAS even lower (range: 0.04 to 0.22). NVP (range: 0.15 to 0.24) and EFV (range: 0.20 to 0.36) drug concentration also showed poor agreement with MEMS^®^. Caregiver-reported missed doses of three-day, seven-day and 30-day recall significantly increased in agreement with MEMS^®^ from month 1 to month 2. Agreement was inconsistent thereafter, but generally remained higher than at month 1. Compared to other measures, the clinician-administered adherence items had the lowest agreement with MEMS^®^ and did not illustrate a significant improvement in agreement from month 1 to month 2 (range: 0.06 to 0.16). Simple binary correlation matrices between caregiver-reported missed doses revealed high correlations between adherence by different recall periods (three-day, seven-day, and 30-day), particularly for caregiver-reported missed doses in the past three days and in the past 30 days.

**Table 3:** Agreement between multiple measures and dichotomized MEMS^®^

	Kappa statistics					
	
Adherence measure	Visit 1	Visit 2	Visit 3	Visit 4	Visit 5	Visit 6
3-day recall	0.05	0.25**	0.14*	0.14*	0.12*	0.23*
7-day recall	0.11	0.29**	0.21*	0.24**	0.34**	0.29**
30-day recall	0.10	0.33**	0.36**	0.28**	0.37**	0.30**
VAS	0.10	0.21	0.22*	0.08	0.04	0.20*
NVP drug concentration	0.15*	–	–	0.24**	–	–
EFV drug concentration	0.36**	–	–	0.20	–	–
AMPATH clinical encounter form	0.11*	0.13*	0.12*	0.06	0.16*	0.12*

* *p*<0.05

** *p*≤0.001.

### Predictors of adherence and treatment interruptions

In univariable analyses, factors associated with dichotomized MEMS^®^ adherence (Table 4) and treatment interruptions (Table 5) were reported by caregivers as: problems in the community, problems with giving the child medicines, medicines making the child sick, forgetting to give the medicines, giving late doses and missing doses. In repeated-measures logistic regression models, only caregiver-reported missed doses in the last 30 days (OR 1.25, 95% CI 1.14–1.39), late doses in the past seven days (OR 1.14, 95% CI 1.05–1.22) and caregiver-reported problems with getting the child to take ART (OR 1.10, 95% CI 1.01–1.20) were significantly associated with dichotomized MEMS^®^. Caregiver-reported difficulties related to community-level factors (OR 1.14, 95% CI 1.02–1.27) and medication side effects (OR 1.12, 95% CI 1.01–1.25) were both significantly associated with treatment interruptions of ≥48 hours.

**Table 4:** Items associated with dichotomized MEMS^®^ non-adherence

	Odds ratios (95% confidence interval)	
	
Caregiver-reported factors	Univariable model	Multivariable model^a^
Forget to keep time when giving the medicines	1.07 (1.02–1.14)	1.05 (0.99–1.11)
Problems getting the child to take the medicines	1.15 (1.05–1.26)	1.09 (1.01–1.20)
Problems giving the child medicine because the child does not know reason for medicines. (i.e. child does not know HIV status)	1.18 (1.02–1.37)	1.13 (0.97–1.32)
Missed doses in past 3 days	1.19 (1.07–1.32)	0.93 (0.81–1.06)
Missed doses in past 30 days	1.27 (1.17–1.39)	1.22 (1.09–1.38)
Missed all doses in a day in past 7 days	1.15 (1.04–1.25)	1.00 (0.87–1.15)
Missed one dose in a day in past 7 days	1.21 (1.11–1.32)	1.10 (0.97–1.24)
Took a dose more than one hour late in past 7 days	1.19 (1.10–1.28)	1.13 (1.06–1.22)

a Multivariable models adjusted for baseline gender, age (years) and duration on ART (years).

**Table 5:** Items associated with MEMS^®^ treatment interruptions of ≥48 hours

	Odds ratios (95% confidence interval)	
	
Caregiver-reported factors	Univariable model	Multivariable model^a^
Problems keeping time when giving the medicines	1.06 (1.01–1.11)	1.04 (0.99–1.10)
Problems getting the child to take the medicines	1.11 (1.03–1.21)	1.05 (0.96–1.16)
Medicines have made the child sick or ill	1.10 (1.01–1.20)	1.11 (1.01–1.24)
Difficulties in the community causing missed dose	0.94 (0.85–1.05)	1.13 (1.01–1.27)
Missed doses in past 30 days	1.09 (1.02–1.17)	1.05 (0.97–1.14)
Missed all doses in a day in past 7 days	1.13 (1.02–1.24)	1.05 (0.93–1.17)
Took a dose more than one hour late in past 7 days	1.08 (1.02–1.15)	1.05 (0.98–1.11)

a Multivariable models adjusted for baseline gender, age (years) and duration on ART (years).

## Discussion

In this cohort, adherence estimates by MEMS^®^, caregiver reports and plasma drug concentrations varied widely. MEMS^®^ revealed high median rates of adherence (96% doses taken) for this cohort, but lower rates of mean adherence (87% of doses taken) and treatment interruptions were common. Caregiver-reported seven-day and 30-day recall of any missed doses to study personnel best correlated with MEMS^®^ adherence, although no adherence measure showed good agreement with MEMS^®^ and caregiver-reported missed doses to clinicians during routine clinic visits had the poorest agreement. Only the caregiver-reported problems with giving the child ART, medication side effects and difficulties related to community-level factors (particularly HIV-related stigma and discrimination) were significantly associated with MEMS^®^ adherence. The relationship between caregiver-level HIV-related stigma and paediatric adherence deserves further investigation, as a large study among African adults found that individuals who reported more perceived HIV stigma were more likely to report non-adherence to therapy [32].

Caregiver report is one of the most commonly used adherence assessment methods for HIV-infected children in resource-limited settings, but it likely overestimates adherence to ART compared to other, more objective measures like pill counts, pharmacy refill and electronic dose monitoring [13]. We also found caregiver-reported adherence to be significantly higher than adherence by MEMS^®^, and there was generally poor agreement between caregiver-reported missed doses and MEMS^®^. Our findings are consistent with work in Zambia that found relatively poor agreement between MEMS^®^ and other adherence measures like caregiver-reported missed doses, VAS and pill counts [33]. Other studies also suggest that caregiver reports overestimate their child's adherence to ART and are not a valid adherence assessment strategy [34–37].

The longitudinal nature of this study allowed us to detect changes in adherence. We found significantly improved MEMS^®^ adherence from month 1 to month 2, which was sustained over the course of the study and is consistent with a similar study in Zambia [33]. Our study was not designed to measure an intervention effect, but the significant change suggests that some aspect of the study procedures may have improved adherence in this cohort. Further work is needed to evaluate individual components of the study procedures (e.g. discussing adherence and barriers with study staff, reviewing MEMS^®^ feedback, having medications in bottles with MEMS^®^ etc.) as an adherence intervention. Using feedback from MEMS^®^ as part of adherence counselling has been shown to be effective in improving adherence in randomized controlled trials among adults living with HIV [38,39]; however, there are few data available for children. Caregivers generally reported more missed doses to study personnel after month 1, despite significantly higher MEMS^®^ adherence. This may suggest that caregivers became more comfortable reporting non-adherence to study personnel over time or that the use of MEMS^®^ feedback encouraged more reporting of non-adherence because caregivers knew that the study team could see the number and timing of missed doses by MEMS^®^. We hypothesize that the caregiver-reported adherence to study staff became less biased and more accurate as the study progressed. This trend was not evident in caregiver-reported missed doses to clinicians, suggesting that adherence assessments outside the patient-provider relationship (e.g. using adherence counsellors or case managers) may yield more accurate reporting of non-adherence for children.

The frequent treatment interruptions of ≥48 hours in this cohort were concerning as interruptions increase risks of drug resistance and viral rebound [40–42]. Unplanned treatment interruptions may be more likely in LMICs due to barriers like inconsistent drug supplies, the financial costs of drugs and transportation to clinic, food insecurity and HIV stigma [19]. AMPATH did not have any major pharmacy stock-outs during the study period; however, malnutrition and poverty were high in this cohort. Previous qualitative work among caregivers of HIV-infected children in this setting suggested that HIV stigma and HIV disclosure pose significant challenges to ART adherence [43]. The association between caregiver-reported community-level factors (e.g. stigma) and treatment interruptions in this study supports the idea that social factors impact adherence. Children's knowledge of their own HIV status may impact adherence, but we did not find a significant association between disclosure and adherence. At least 25% of caregivers reported giving a late dose in the past week at monthly visits, but late doses were not associated with treatment interruptions, suggesting that different factors may affect interruptions versus delays. For example, in previous qualitative work in this setting, we found that children experience treatment interruptions when traveling over the weekend, whereas delays are experienced when caregivers arrive home late at night from work or visitors are in the home [43].

Collecting plasma drug concentrations was a unique aspect of this study and is not often available in this setting. We found that significant numbers of children had sub-therapeutic and supra-therapeutic drug levels. This is concerning as sub-therapeutic levels are associated with drug resistance and virologic failure, while supra-therapeutic levels are associated with increased frequency and severity of side effects in adults living with HIV [44–48]. Children on EFV were more likely to have sub-therapeutic levels than children on NVP, whereas children on NVP were more likely to have supra-therapeutic levels; in fact, more than half of children on NVP had supra-therapeutic drug levels. Drug concentrations did not correlate well with MEMS^®^ adherence, which may be due to several factors. First, MEMS^®^ adherence was calculated for all doses taken between study visits (approximately one month), whereas drug concentrations only indicate adherence within hours to days. Drug concentrations would likely correlate better with MEMS^®^ if MEMS^®^ were restricted to doses taken in the 2-3 days prior. Second, drug concentrations may be influenced by the dearth of pharmacokinetic data for antiretroviral drugs in children, especially in sub-Saharan Africa [49,50]. AMPATH follows standard paediatric dosing recommendations, but it is possible that these dosing guidelines are not optimal and lead to sub-therapeutic or supra-therapeutic drug concentrations, even if the patient is adherent. Inappropriate drug levels may reflect problems with dosing recommendations more than adherence difficulties. A study among children on EFV-based regimens in South Africa found that 40% had sub-therapeutic drug levels (<1.0 µg/mL), while another study in South Africa reported median sub-therapeutic levels of EFV in 17% of children at one, three and six months of follow-up [51,52]. The proportion of children in our cohort with supra-therapeutic drug levels also deserves further consideration, particularly because caregiver-reported medication side effects were significantly associated with treatment interruptions. Studies in Malawi, Zambia and Germany have reported high rates of supra-therapeutic drug levels in children, although not as high as those found in our study [53,54]. More pharmacokinetic studies among African paediatric populations are needed to ensure that dosing recommendations are appropriate.

Several limitations of this study deserve consideration. First, this study employed convenience sampling that introduces potential selection bias and, in this case, might have led to children who had excellent adherence or very low adherence being more likely to enrol. Regardless of baseline adherence levels and whether they were higher or lower than those of the general AMPATH paediatric population, we still should have been able to detect significant associations between adherence and various clinical, demographic and social factors. Second, the intensive study procedures likely affected adherence over the course of the study; however, we could not evaluate these effects. Third, electronic drug monitoring is typically reserved for research settings, as the technology is often too expensive for routine monitoring. Furthermore, MEMS^®^ has important limitations (e.g. patients removing more than one dose of medicines or switching between bottles) that have not been adequately explored in this setting [55,56]. Fourth, we did not have access to viral load testing for this cohort of patients. Virological suppression is considered the most important outcome of adherence to therapy [57]; however, due to funding constraints, viral loads were unavailable. Finally, our sample of 191 children was relatively small, and follow-up time was relatively short. Children were on first-line ART for a mean of 2.3 years and most were entering early adolescence, so issues of treatment fatigue and disclosure of HIV status may present additional barriers to adherence in the coming years [58]. Furthermore, this study took place at the largest, most urban clinic within AMPATH, and so our findings may not be generalizable for smaller or more rural clinics. Nonetheless, this sample provides detailed adherence measurements for a sub-Saharan African paediatric population and provides preliminary support for validating routine adherence monitoring in paediatric populations in LMICs, as well as pointing to electronic monitoring with feedback as a potential adherence intervention.

## Conclusions

We found that caregiver-reported missed doses overestimate children's adherence to ART compared to electronic dose monitoring. Despite high rates of adherence by caregiver report, missed and late doses, treatment interruptions of more than 48 hours and sub-therapeutic drug levels were common.

## Supplementary Material

Measuring adherence to antiretroviral therapy in children and adolescents in western Kenya
