# Supplementary material for: Measuring adherence to antiretroviral therapy in children and adolescents in western Kenya
Source: J Int AIDS Soc. 2014 Nov 25;17(1):19227. doi: 10.7448/IAS.17.1.19227 (PMC4245448; doi:10.7448/IAS.17.1.19227)

AMPATH CLINICS
Comprehensive ART Adherence Measurement for Paediatrics (CAMP) – Caregiver Evaluation

**Patient Name(s):**		**Date:**
**Patient AMPATH ID:**		**Patient Date of Birth:**
**Patient Sex:**	**Patient Age:**	**Relationship of person completing form to child:**
**Parent/Guardian Name:**		**Study ID:**
**Clinic Location:** MTRH Module: □ 1 □ 2 □ 3 □ 4 □ Amukura □ Burnt Forest □ Busia □ Chulaimbo □ Iten □ Kabarnet □ Kapenguria □ Khunyangu □ Kitale □ Mosoriot □ Mt. Elgon □ Naitiri □ Port Victoria □ Teso □ Turbo □ Webuye □ Other:		
**Date of Study Start:**		**When did child start on ART?**

I am going to ask you some questions about your child [child's name] and his/her medicines. This questionnaire is designed to help us understand how it is for you both to do what you have been asked to do with the medicines. Please answer these questions as best you can. Your answers to these questions will not keep you from getting the medicine you need. The questions just help us assist you in taking the medicine correctly.

Medication Description Questions

1. What are the medicines [child's NAME] is supposed to be taking for HIV infection? *Ask the caregiver to show you the medicines. Have the caregiver explain how much is given and when. If they do not know names, record whatever the caregiver says to describe the medicine.*

Medication Name | Amount of Medication to be Given | Times Given

______________________ □ Could not name ______________________

______________________ □ Could not name ______________________

______________________ □ Could not name ______________________

______________________ □ Could not name ______________________

______________________ □ Could not name ______________________

______________________ □ Could not name ______________________

______________________ □ Could not name ______________________

2. Any other medicines given to [child's NAME]? If yes, which ones?

Medication Name | Amount of Medication to be Given | Times Given

______________________ □ Could not name ______________________

______________________ □ Could not name ______________________

______________________ □ Could not name ______________________

______________________ □ Could not name ______________________

3. What herbals, teas, or traditional medicines is [child's NAME] using?

____

____

4. Is [child's NAME] using any other medications from other doctors, clinics or hospitals? If yes, which ones?

Medication Name | Amount of Medication to be Given | Times Given

______________________ □ Could not name ______________________

______________________ □ Could not name ______________________

5. What else have you been doing or using to help the child become strong or healthy?

____

____

AMPATH CLINICS

Comprehensive ART Adherence Measurement for Paediatrics (CAMP) – Caregiver Evaluation

6. Who gives [name] his/her medicines? *(tick all that apply)* □ Mother □ Father □ Guardian □ Relative who lives in home □ Relative who lives outside of home □ Neighbor □ Sibling □ House help □ Child takes meds themselves □ Other (specify) ______________________

7. Does anyone besides you know that [name] takes these medicines? □ Yes □ No
If yes, how many people know?_______________
Who knows? (specify)_______________________
If no, why not? (specify)_______________________
____

8. In an average week, how many days of the week are you the one who gives the child medicines?
□**Morning doses** □ 0 □ 1 □ 2 □ 3 □ 4 □ 5 □ 6 □7
□**Evening doses** □ 0 □ 1 □ 2 □ 3 □ 4 □ 5 □ 6 □ 7

9. Does the child know that he/she is taking the medicines for HIV? □ Yes □ No

Many parents and caregivers tell us that they sometimes have problems with giving the child medicines every day or at the right time. There are many reasons for families to struggle with the medicines. Many parents just forget when they are too busy or they do not give the medicines when they do not have food.

10. Do you ever just forget to give the medicines when you are busy?
□ Yes □ No
If yes, how often?
□ Many times □ Some times
□ Occasionally ____times in a week

11. Do you ever forget to keep time in giving the medicines?
□ Yes □ No
If yes, how often?
□ Many times □ Some times
□ Occasionally ____times in a week
When? □ Mornings □ Evenings
□Weekends □ Weekdays □ Other:
____

12. Do you ever have problems keeping time with the medicines?
□ Yes □ No
If yes, how often?
□ Many times □ Some times
□ Occasionally ____times in a week
When? □ Mornings □ Evenings
□Weekends □ Weekdays □
Other:_________________

13. Do you ever not give the medicines because you do not want to give them in front of other people?
□ Yes □ No
If yes, how often?
□ Many times □ Some times
□ Occasionally
___times in a week

14. Do you ever delay giving the medicines because you do not want to give them in front of other people?
□ Yes □ No
If yes, how often?
□ Many times □ Some times
□ Occasionally
____times in a week

15. Are there times when you do not have enough food for your family?
□ Yes □ No

How many meals do you miss in a week?
____meals in a week

How many meals does your child miss in a week?
____meals in a week

16. Do you ever not give the child the medicines because you do not have food to give with the medicines?
□ Yes □ No
If yes, how often?
□ Many times □ Some times
□ Occasionally ____times in a week

17. Are you currently enrolled in AMPATH nutrition program?
□Yes □No

18. Do you ever have problems with getting your child to take the medicines?
□ Yes □ No
If yes, how often?
□ Many times □ Some times
□ Occasionally ____times in a week

What problems does child raise?
____
____
__

19. Do you ever have problems with giving the medicines because the child does not know why they are taking them?
□ Yes □ No
If yes, how often?
□ Many times □ Some times
□ Occasionally
___times in a week

20. Have the medicines ever made the child sick or ill? □Yes □No
If yes: □ Vomiting □ Rash □ Anemia
□ Sleep problem □ Liver problem
□ Other:_____________________
If yes, why do you think the child became ill? □ Medicines too strong
□ Side effect of medicines
□ Did not take with food
□ Child not used to medicine
□ Other reason (specify)__________
____

21. Did your child miss any doses yesterday? □Yes □No
□ Don't know

22. How many doses of medicine has your child missed in the last 3 days?
(write number) _________
□ Don't know

23. How many doses of medicine has your child missed in the last month? _________________
□ Don't know

24. Some families tell us that their child worries them or makes it difficult to give them the medicines. Has your child [name] not taken medicines for any of these reasons:
□ He/she does not know why taking the medicines or keeps asking questions about the medicines
□ He/she did not understand the medication instructions □ He/she forgot to take medicine
□ He/she was playing or at school or work □ He/she refused to take medicine
□ He/she felt ill or was vomiting □ He/she felt better
□ He/she does not want others to see the medicines □ He/she believes medicine does not help
□ He/she had harm or side effects caused by the drugs □ Has problems with 1 formulation (tablets, liquids)
□ Finds medicines too bitter □ He/she is tired of taking the medicines
□ Can't take without food □ None of the above
□ Other (specify:______________________________________

25. Sometimes, a child does not take their medicines every day or at the same time every day because of difficulties for the caregiver. I am going to read a list of issues that may be problems for you as a caregiver in having the child take the medicines. Stop me when you hear a problem mentioned that applies to you or the child's caregiver. I [or the caregiver]:
□ I had difficulty with reading instructions □ I was afraid of side effects on child
□ I did not understand the medication instructions □ I thought other matters were more urgent
□ I thought treatment was completed □ I was away from home (work, field, etc.)
□ I was not always around with the child □ I was discouraged or losing hope
□ I was taking alcohol or other drugs □ There were frequent changes in caregivers
□ I did not want others to see □ Caregiver being too busy and forgetting
□ I had trouble with timing or giving the doses on time □ I was not aware of child's status
□ I did not think the drugs were helping □ I wanted to try another treatment or prayers
□ I thought child needed a break from the medicines □ None of the above
□ Other (specify) ___________________________

26. Sometimes, children do not take their medicines because of difficulties within the community. Have any difficulties in the community caused your child to miss taking their medicines? Stop me when you hear a problem mentioned that applies to you:
□ I was unable to explain why the child taking medicines □ I did not want the child to be seen taking medicine
□ I was being discouraged by neighbors/friends/family □ I feared discrimination and isolation
□ Child in school and I did not want to remove from school □ Others did not believe medicines are needed
□ I did not receive help from neighbors/friends/family □ Other: (specify)___________________________
□ Could not get to clinic without others wondering □ None of the above

27. Sometimes, problems at the clinic make it difficult for families to give these medicines every day. Have any of these things been a problem for you:
□ The clinic staff didn't explain well enough how to give or take the medicine or did not write instructions
□ The clinic staff seemed to have a negative/judgmental attitude about the medicines
□ The clinic staff made you feel harassed
□ There was no money to purchase medicine (if not offered at AMPATH)
□ The medicine was not available in the pharmacy. ***Which medicine?*** □ ARVs □ Septrin □ Other (include abx)
□ Other (specify) ___________________________________□ None of the above

28. When children are sick, families often try other forms of treatment in addition to or in place of the ARVs. Is your child currently going for any of these other types of treatment: (Specify *tick all that apply*)
□ Herbal (including leaves, stems, roots) □ Teas □ Chinese □ Prayers for healing □ South African supplements
□ Witchcraft □ Cutting □ Other: (specify) ____________________

29. At times, families have difficulties with other matters related to the medicines. Have any of these things made it difficult for your child to take the medicines everyday or at the right time?
□ Too little/no food to give with medicine □ Ran out of medicine before clinic appointment
□ Pouring of medicines □ Family refused medication
□ Nobody to administer medication □ No clean water to use with medicines
□ Needing to hide medicines □ Delaying doses of medicines
□ No money for transport to clinic □ No transport to clinic available
□ Other (specify) _________________________________ □ None of the above

30. In general how do feel about taking medicine? □ I am willing to take medicine □ I dislike taking medicine, but I take it when I need it □ I use herbs instead of taking pills □ I never take medicine for any reason

31. Imagine I could give you 5 cows now OR I could give you 8 cows in 5 years. This is not a real situation; this is a hypothetical situation to imagine. Which would you prefer? □ 5 cows now □ 8 cows in 5 years

32. We want to know whether you agree or disagree with this statement: "I will sometimes give something up now so that I will get something better in my future." An example is: "I will not slaughter my cow for meat this year so that my cow could have a calf next year, and then I could slaughter 2 cows." Do you:
□ Strongly disagree □ Disagree □ Neither agree nor disagree □ Agree □ Strongly agree

AMPATH CLINICS
Comprehensive ART Adherence Measurement for Paediatrics (CAMP) – Caregiver Evaluation

33. Everyone misses taking their medication sometimes for various reasons. Do you have any trouble giving the child their medicines? □ Yes □ No **If yes, how often?** □ Many times □ Some times □ Occasionally

34. In the past week,
a. How many days were you with the child? □0 □1 □2 □3 □4 □5 □6 □7
b. On how many days did the child miss at least one dose? □0 □1 □2 □3 □4 □5 □6 □7
c. On how many days did the child take a dose more than an hour late? □0 □1 □2 □3 □4 □5 □6 □7
d. On how many days did the child miss **all** of his/her doses? □0 □1 □2 □3 □4 □5 □6 □7
e. How many doses did the child miss altogether? _______
f. How many extra doses or syringes of medicine did the child take? _______

35. How many people usually live in your household or are staying with you now? ________

36. How many children under 5 years of age live in your household?________

37. How many people in your household take medicines for HIV? __________________

38. Who else in the household takes medicines for HIV?
____
____

39. Who do you and this child stay with? *(Tick all that apply)*
□ No one (stay alone) □ Child's Parents
□ Caregiver's Spouse □ Child's Grandparents
□ Caregiver's Partner □ Child's Uncle/ Auntie/cousins
□ Other children □ Friends

40. Which of these people know the child takes medicines?*(Tick all that apply)*
□ No one (stay alone) □ Child's Parents
□ Caregiver's Spouse □ Child's Grandparents
□ Caregiver's Partner □ Child's Uncle/Auntie/Cousins
□ Other children □ Friends

41. Where do you get your water for drinking?
□ Piped (outside)
□ Piped (in home)
□ Borehole
□ River/stream/lake
□ Other ___________

42. Do you boil your drinking water?
□ Yes, always
□ Yes, sometimes
□ No
If no, use other treatment?
□ Yes, always
□ Yes, sometimes
□ No

43. Are you employed outside the home?
□ Yes □ No
If yes:
□ Full-time
□ Part-time
□ Casual
□Self employed

44. How long does it take you to travel to clinic?
□ ≤ 30 minutes
□ 30min-1hr
□ >1hr but <2hr
□ >2hr but <3hr
□ >3 hrs

45. How much do you pay for transport to come to clinic (one way)?
_______ Ksh

46. Do you have any difficulties with transport to clinic?
□ Yes □ No **If yes, how often?** □ Many times □ Some times □ Occasionally
What problem? □ lack of money □ lack of means □ lack of time □ Other ___________

Many families try to take their pills around the same time or with the same activity every day so that they won't forget to take the medicines…..

47. Is there something that you are currently doing that helps to remind you to give the child his or her medicines at the same time every day? □ Yes □ No

If yes, what helps to remind you? □ Phone □ Watch □ Radio □ Taking medicines at meal times □ Sun □ Others in house take medicines together □ Other: ___________________________________

48. ***Put a cross on the line below at the point showing your best guess about how much of each drug the child has taken in the last month for both the morning and the evening doses. For example, putting a mark on the very left for the morning picture means that the child has missed all of their doses in the morning every day. Putting a mark on the right means that they have taken all of their morning doses every day. A mark in the middle means that they have taken half of their morning doses. Please do this for morning and evening.***

Morning Doses:

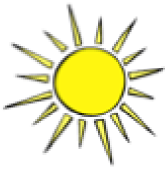

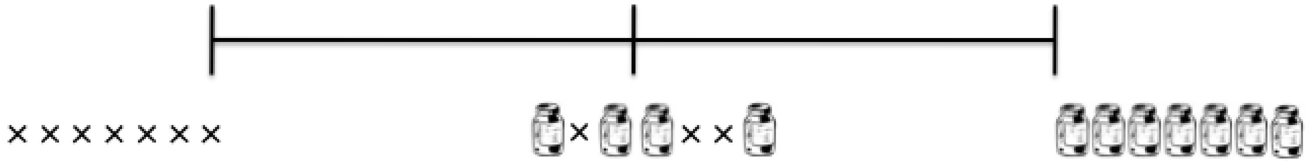

Evening Doses:

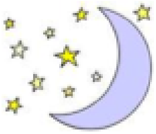

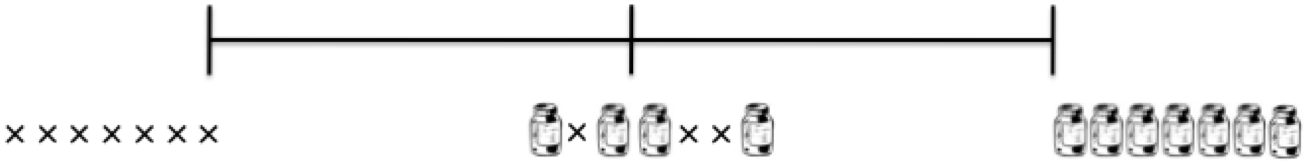

Supplement: Measuring adherence to antiretroviral therapy in children and adolescents in western Kenya [file JIAS-17-19227-s001.pdf]
